# Comparison of Frontal Sinus Dimensions with Different Skeletal Classes and Vertical Patterns: A Retrospective Study

**DOI:** 10.3390/healthcare13111310

**Published:** 2025-05-31

**Authors:** Alessandro Nota, Iuliia Kashtelianska, Francesco Manfredi Monticciolo, Laura Pittari, Simona Tecco, Attilio Castaldo

**Affiliations:** 1 Dental School, Vita-Salute San Raffaele University and IRCCS San Raffaele Hospital, 20132 Milan, Italy; nota.alessandro@hsr.it (A.N.); francesco.manfredi.monticciolo@gmail.com (F.M.M.); laura_pittari@hotmail.it (L.P.); 2 Dental School, University of Trieste, 34127 Trieste, Italy; gkashtelianska@gmail.com; 3 MeSVA, University of L’Aquila, 24100 L’Aquila, Italy; 4 Department of Orthodontics, University of Trieste, 34127 Trieste, Italy; attiliocastaldo@gmail.com; 5 Department of Orthodontics, Virginia Commonwealth University, Richmond, VA 23173, USA

**Keywords:** angle class III malocclusion, mandibular prognathism, growth and development, frontal sinus, cranial sinuses

## Abstract

Background: The aim of this work is to compare the size of the frontal sinus and the different skeletal classes and divergence patterns of the subjects. Methods: This study retrospectively includes lateral radiographs performed on a total of 200 adults (78 M, 122 F; mean age 29.2 ± 8.0 years). Subject inclusion criteria were an age of 18–45 years, presence of both frontal sinuses, and good general health with no hormonal system disorders that may affect the growth and pneumatization of the frontal sinuses. Four different parameters of the frontal sinus were evaluated: length, width, perimeter, and area. In order to calculate the variables inherent to the sagittal and vertical skeletal pattern, two independent nominal variables were considered: skeletal class (ANB°) and mandibular divergence (SN^MP°). Results: The statistical analysis showed that there is a statistically significant difference between the frontal sinus dimension and the three skeletal classes. Subjects in group 3 presented significantly higher mean dimensional values. In the analysis of sinus size variables with skeletal divergence, significant results were found for the width value, which resulted in higher average values in group c. Conclusions: The present study shows a statistically significant difference in frontal sinus size among different skeletal classes and divergence patterns. This result suggests that, in future studies, it should be analyzed whether the dimensional analysis of the frontal sinus could be associated with skeletal class III malocclusion.

## 1. Introduction

The frontal sinus is a cavity located in the frontal bone and is part of the paranasal sinuses of the cranio-maxillofacial complex [1]. The dimensional development of the paranasal sinuses significantly contributes to establishing the final morphology and the intermaxillary skeletal relationship. The frontal sinuses have unique characteristics in each individual. In fact, these can be used for identity recognition in forensic medicine. In some individuals, the frontal sinuses may not develop. This condition is called “frontal sinus aplasia” [2,3]. In particular, given the position of the frontal sinus and its anatomical relationship with the anterior cranial base and with the maxillary nasal complex, the pneumatization of the frontal sinus assumes a main role in facial development [2,3,4]. Evidence shows that frontal sinus dimensions have a multifactorial origin and are related to genetic factors and weather conditions [4,5,6]. The frontal sinus is not visible at birth [7,8,9]; with the pneumatization process, it becomes radiographically evident with age [8,9,10]. According to Brown et al. [11], the main development of the frontal sinus occurs at the age of 15.5 years for male subjects and at the age of 13 years for female subjects. Some studies claim that the frontal sinus becomes radiographically evident, on average, 1.4 years after the peak growth spurt [2,3,4,5,6,7].

Yassenia et al. [10] found that the frontal sinus reaches its largest dimensional increment with a growth peak about 1 year after the puberal body height peak. McCann’s study states that the development of the frontal sinuses can also affect facial growth.

This was deduced following an experiment carried out on chicken embryos where it was observed that the surgical removal of the fronto-nasal structures greatly reduced the development of the middle part of the splanchnocranium [11]. Some studies found that an oversized mandible and class III skeletal pattern and an oversized frontal sinus can be associated with pathological conditions like acromegalia [9], but this association was also found in patients with normal systemic conditions [3,10,12].

Several studies have shown that the frontal sinus extension can be associated with the skeletal growth pattern [3,4,5,6,7,8,9,10,11,12].

Contrastingly, a study by Husamettin carried out using 189 latero-lateral teleradiographs indicated that there was no correlation between the parameters examined, except for those relating to class II, which appeared to be greater in women [13]. Several studies have shown that the frontal sinus extension can be associated with the skeletal growth pattern [3,4,5,6,7,8,9,10,11,12,13,14,15].

The growth patterns of the mandible and maxilla are considered as essential elements to determine the prognosis of, and consequently the treatment timing for, malocclusions [16].

Considering the previous literature, the role played by the frontal sinuses in growth patterns and facial symmetry suggests the evaluation, among other things, of the morphology of the frontal sinus in orthodontic diagnosis. The aim of this work is therefore to compare the size of the frontal sinus among the different skeletal classes and divergence patterns of the subjects.

## 2. Materials and Methods

The aim of this work is to analyze the differences in the size of the frontal sinus among subjects with different skeletal classes and divergence patterns. The present study retrospectively includes lateral radiographs performed on a total of 200 adults (78 males, 122 females; mean age 29.2 ± 8.0 years), patients of the Dental Clinic of the Department of Life Science at the University of Trieste. The radiographs were acquired over the period between 1 January 2007 and 30 July 2020 and were prescribed by the referring clinician for diagnostic evaluation prior to orthodontic treatment.

The following inclusion criteria were applied to the sample: age between 18 and 45 years at the time of radiologic examination, presence of both frontal sinuses by antero-posterior radiography, and good general health with no hormonal system disorders that may affect the growth and pneumatization of the frontal sinuses.

All the subjects of the radiographs included in the study had previously given informed consent for their personal data to be used for educational and scientific purposes, and the protocol was approved by the Ethics Committee of the University of Trieste with document number 104 at the meeting of 11 May 2020.

### 2.1. Procedures

A dimensional assessment of the frontal sinuses was performed using the digital image-processing computer program ImageJ (version 2.0.0- rc-69/1.52i, National Institutes of Health, Bethesda, MD, USA).

**Figure 1:**
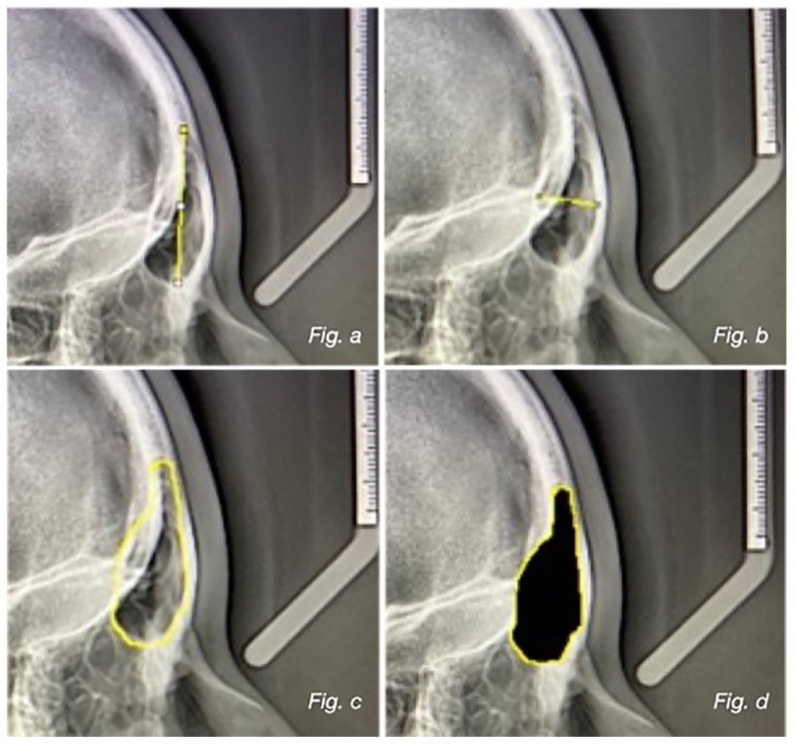
(**a**) Frontal sinus length measured from the highest to the lowest point; (**b**) width of the frontal sinus measured from the most posterior point to the most anterior point; (**c**) perimeter of the frontal sinus represented by the circumferential area; (**d**) area of the frontal sinus calculated in the circumferential zone.

Four different parameters of the frontal sinuses were evaluated and they were measured from the lateral radiograph: frontal sinus length measured from the highest to the lowest point; width of the frontal sinus measured from the most posterior point to the most anterior point; perimeter of the frontal sinus represented by the circumferential area; area of the frontal sinus calculated in the circumferential zone (Figure 1a–d) [17].

In the case of double contours in the frontal sinus image, a midline contour was traced and considered for the analysis.

Then, a cephalometric analysis was performed using the digital software Delta-Dent (version 2.1.0.Ink, Outside Format, Pandino, Italy) in order to calculate the following variables inherent to the sagittal and vertical skeletal pattern.

Two independent nominal variables from the cephalometric analysis were included in the data sheet and analyzed: ANB angle (skeletal class) and SN^MP angle (divergence).

The method error was estimated by repeating the frontal sinus measurement process for 15 randomly selected radiographs. Values from the two measurement sessions were analyzed by applying Dahlberg’s formula, which showed a method error of 0.1 mm.

### 2.2. Statistical Analysis

The data obtained from the dimensional analysis of the frontal sinuses of the 200 patients were descriptively analyzed, stratifying the sample into 3 groups for each independent variable. In particular, according to the ANB angle reference values, the sample was divided into 3 groups: group 1 (class I—0 < ANB < 4, n = 74), group 2 (class II—ANB ≥ 4, n = 101), and group 3 (class III—ANB ≤ 0, n = 25).

According to the SN^MP angle reference values, the sample was divided into group a (hyperdivergence—SN^MP ≥ 37, n = 15), group b (hypodivergence—SN^MP ≤ 27, n = 83), and group c (normodivergence—27 < SN^MP < 37, n = 102) according to the mandibular divergence [18].

After confirming a normal distribution of the data by applying the Kolmogorov–Smirnov test, a 2-way ANOVA was applied to assess whether there was a statistically significant difference between the mean values of the frontal sinus size variables of the groups.

In the case of statistically significant differences, Fisher’s LSD post hoc test was subsequently performed to assess the differences between the subgroups.

The sample size of the present study was established by performing an estimation analysis of the number of subjects required to achieve a power of 80% with an alpha error of 0.05 on a sample of preliminary data of the present study, obtaining a minimum number of 196 patients. The actual power of the study evaluated at the end of the analysis was 81%.

## 3. Results

The descriptive statistics of the dimensional variables of the skeletal class and divergence for the whole sample are represented in Table 1, while the results of the statistical analysis of the frontal sinus size variables in relation to different skeletal classes and divergence are shown in Table 2, Table 3 and Table 4.

**Figure 2:**
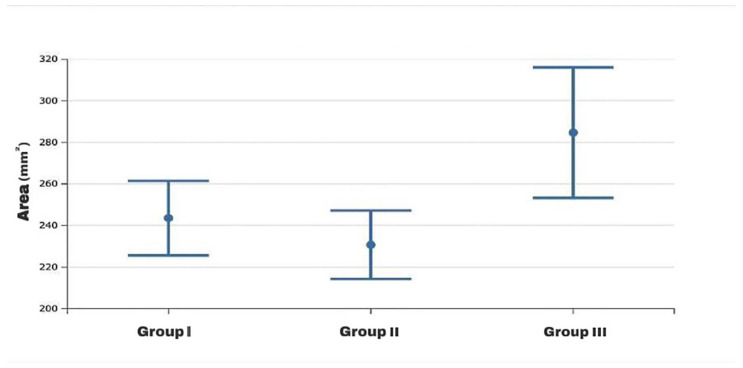
Box plot of area values of the frontal sinus among the study groups.

**Figure 3:**
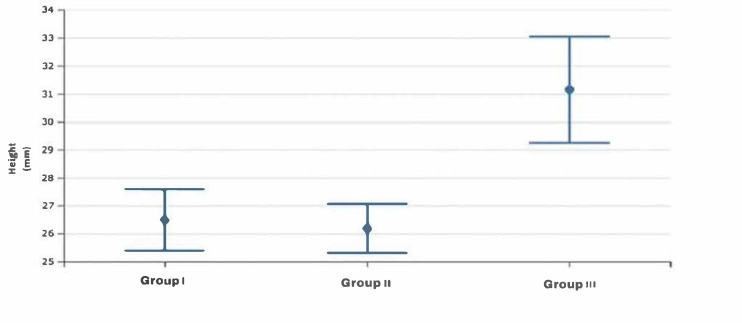
Box plot of height values of the frontal sinus among the study groups.

**Figure 4:**
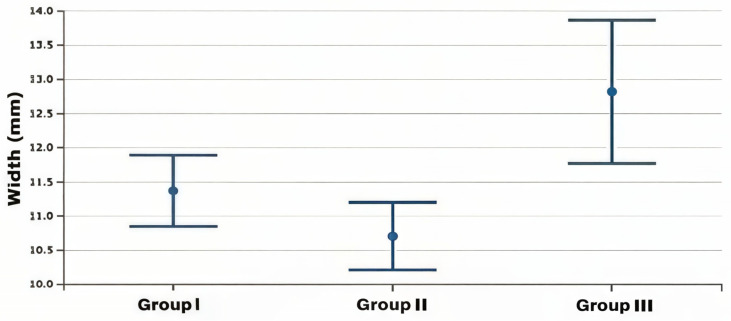
Box plot of width values of the frontal sinus among the study groups.

**Figure 5:**
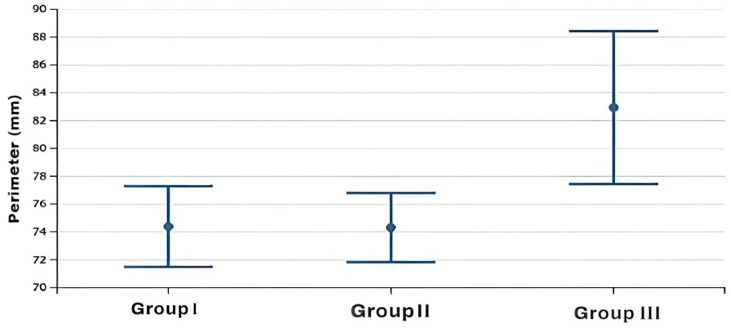
Box plot of perimeter values of the frontal sinus among the study groups.

The statistical two-way ANOVA showed that there were statistically significant differences among the mean values of the frontal sinus size (area, height, width, perimeter) of the three skeletal class groups (*p* < 0.0001 for all variables except area with a *p* = 0.012) (Table 2). In particular, the subjects in group 3, as shown by Fisher’s post hoc test, presented significantly higher mean dimensional values than the other two groups, which, on the contrary, did not present significant differences between them, although the mean dimensional values of the frontal sinuses of group 2 were always lower than those of group 1 (Table 3) (Figure 2, Figure 3, Figure 4 and Figure 5).

**Figure 6:**
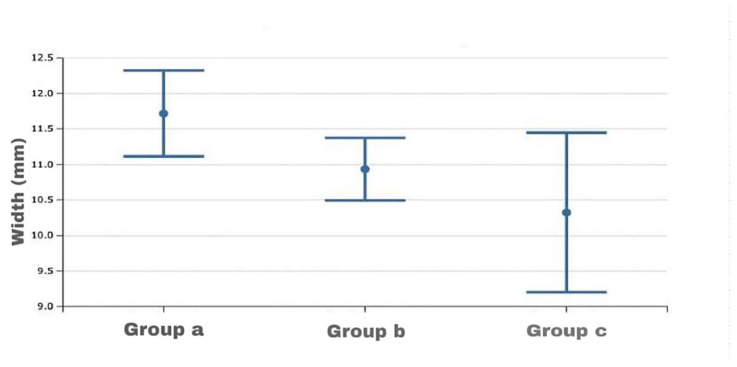
Box plot of width values of the frontal sinus among the divergence study groups.

In the analysis of the frontal sinus size variables’ mean values among the groups selected for maxillary skeletal divergence (group a–group b–group c), significant differences were found only for the width value (*p* = 0.02), with higher average values in group c compared to the other two groups, as shown by Fisher’s post hoc test. There were no significant differences between group a and group b (Table 4) (Figure 6).

## 4. Discussion

The present study analyzed lateral radiographs of a total of 200 patients aged between 18 and 45 years in order to identify a possible difference between frontal sinus size and skeletal class and intermaxillary divergence. The results obtained showed a statistically significant difference in the mean values of frontal sinus size according to the skeletal class. A difference in frontal sinus width was also observed among different skeletal divergence patterns.

In particular, it was observed that subjects in skeletal class III had significantly higher mean frontal sinus size values than subjects in skeletal classes I and II. In this respect, a previous study by Tunca et al. [19] is in agreement with this result, as it showed that an increase in frontal sinus height and length correlated positively with a decrease in ANB angle.

Furthermore, Yassaei et al. also showed the size values of the frontal sinus to be significantly greater in patients in skeletal class III compared to the other two skeletal classes, analyzing the lateral teleradiographs of patients aged 15–20 years [10]. In addition, the size and area of the frontal sinus were found to be associated with the length of the mandibular body and the gonial angle. In contrast, Said et al. argued that frontal sinus size could be used as an indicator of harmonious anterior occlusion but found no significant frontal sinus size differences between subgroups of each skeletal class [20]. Similarly, in contrast to our results, the study by Castaldo et al. carried out on 280 latero-lateral teleradiographs found lower values for frontal sinus size in class III [21]. It could be observed that, in the study by Castaldo et al., the increased dimensions of the frontal sinus in subjects in skeletal class III could be related to a retrognathic maxilla, while, in the present study, the average cephalometric value of the SNB° in skeletal class III was 82.86 and the corresponding value of the SNA° was 80.7, identifying a sample of class III subjects with normomaxillia and hypermandibolia. Castaldo et al. instead evaluated a sample of class III subjects with hypomaxillia and a lower tendency to mandibular prognathism, since class III hypomaxillia is very common in the Caucasian ethnic group. The results of the present study could therefore be in agreement with Rossouw’s consideration that the size of the frontal sinus may be an indicator of mandibular growth [3]. Furthermore, the present study found that greater frontal sinus width is associated with hyperdivergent subjects. 

Previously, Said et al. showed an absence of correlation between the MP-SN° angular value and the area of the frontal sinus, in agreement with the present study, which actually found it only for the value of the width [20]. Also, the study published by Tunca et al. showed the absence of an association between the frontal sinus dimension and skeletal divergence (FMA°, SN-GoGn°) [20]. The present study has some limitations as the dimensional evaluation of the sinuses was carried out on bidimensional X-ray examinations. In the future, it might be appropriate to perform new studies with CBCT to perform a volumetric evaluation. Regarding the results on skeletal class III, it should also be observed that they can only be generalized to a skeletal class III population with pure mandibular progenism/prognathism. Furthermore, in order to have a direct clinical implication, a longitudinal study should be performed to relate the frontal sinus area with the mandibular growth pattern. For these reasons, it would be advisable to carry out future clinical studies evaluating the dimensions of the frontal sinus in three dimensions and also to differentiate the skeletal class III sample according to the presence of a skeletal component of hypomaxillia rather than hypermandibolia.

## 5. Conclusions

The present study shows a statistically significant difference in frontal sinus size among different skeletal classes and divergence patterns. In particular, subjects in skeletal class III with mandibular prominence seem to present greater values for frontal sinus dimensions. This result suggests that, in future studies, it should be analyzed whether the dimensional analysis of the frontal sinus could be associated with skeletal class III malocclusion.

## Figures and Tables

**Table 1:** Descriptive analysis of the cephalometric values analyzed.

Total Sample		
	Mean	SD
**SNA°**	82.41	4.12
**SNB°**	78.70	4.45
**ANB°**	3.73	3.26
**SN-MP°**	32.04	7.37

**Table 2:** Statistical analysis of frontal sinus size variables in relation to different skeletal patterns and classes (2-way ANOVA).

Factor	Group	Mean	DS	*p*-Value
*Area*				
**Class**	1	243.57	77.21	0.012
	2	230.74	83.34	
	3	284.70	76.11	
**Divergence**	a	237.37	77.70	n.s
	b	247.54	88.54	
	c	238.63	76.81	
*Height*				
**Class**	1	26.49	4.71	<0.0001
	2	26.19	4.42	
	3	31.16	4.60	
**Divergence**	a	26.32	3.69	n.s.
	b	27.08	5.17	
	c	26.89	4.68	
*Width*				
**Class**	1	11.37	2.24	<0.0001
	2	10.71	2.51	
	3	12.82	2.54	
**Divergence**	a	10.33	2.03	0.02
	b	11.74	2.79	
	c	10.93	2.23	
*Perimeter*				
**Class**	1	26.49	4.71	<0.0001
	2	26.19	4.42	
	3	31.16	4.60	
**Divergence**	a	26.32	3.69	n.s.
	b	27.08	5.17	
	c	26.89	4.68	

**Table 3:** Fisher’s LSD. Post hoc statistical analysis of frontal sinus size variables in relation to skeletal classes.

Fisher’s LSD		
Area		
Comparison	Difference	***p***-value
1 vs. 2	12.83	n.s
1 vs. 3	−41.13	0.029
2 vs. 3	−53.96	0.003
Height		
Comparison	Difference	*p*-value
1 vs. 2	0.30	n.s.
1 vs. 3	−4.67	<0.0001
2 vs. 3	−4.97	<0.0001
Width		
Comparison	Difference	*p*-value
1 vs. 2	0.66	n.s.
1 vs. 3	−1.45	0.009
2 vs. 3	−2.11	<0.0001
Perimeter		
Comparison	Difference	*p*-value
1 vs. 2	0.08	n.s.
1 vs. 3	−8.55	0.004
2 vs. 3	−8.62	<0.0001

**Table 4:** Fisher’s LSD. Post hoc statistical analysis of frontal sinus size variables in relation to intermaxillary divergence.

Fisher’s LSD		
Width		
Comparison	Difference	*p*-value
b vs. c	0.78	0.033
b vs. a	1.39	0.045
c vs. a	0.61	n.s.

## Data Availability

The data that support the findings of this study are available from the University of Trieste but restrictions are applied to the availability of these data, which were used under license for the current study, and so they are not publicly available. Data are, however, available from the authors upon reasonable request.

## Abbreviations

The following abbreviations are used in this manuscript:

**95% CI**	95% confidence interval
**Fisher LSD**	Fisher’s Least Significant Difference
